# Vision-language models for human motion understanding: Lessons from stroke rehabilitation

**DOI:** 10.1371/journal.pdig.0001506

**Published:** 2026-07-06

**Authors:** Victor Li, Naveenraj Kamalakannan, Avinash Parnandi, Heidi Schambra, Carlos Fernandez-Granda

**Affiliations:** 1 Center for Data Science, New York University, New York, New York, United States of America; 2 Tandon School of Engineering, New York University, Brooklyn, New York, United States of America; 3 VitalConnect, San Jose, California, United States of America; 4 Department of Neurology, NYU Grossman School of Medicine, New York, New York, United States of America; 5 Department of Rehabilitation Medicine, NYU Grossman School of Medicine, New York, New York, United States of America; 6 Courant Institute of Mathematical Sciences, New York University, New York, New York, United States of America; Delft University of Technology: Technische Universiteit Delft, NETHERLANDS, KINGDOM OF THE

## Abstract

Vision–language models (VLMs) have demonstrated remarkable performance across a wide range of computer-vision tasks, sparking interest in their potential for digital health applications. Here, we apply VLMs to two fundamental challenges in data-driven stroke rehabilitation: automatic quantification of rehabilitation dose and impairment from videos. We formulate these problems as motion-identification tasks, which can be addressed using VLMs. We evaluate our proposed framework on 20 healthy controls and 51 stroke survivors. Our results show that current VLMs lack the fine-grained motion understanding required for precise quantification: dose estimates are comparable to a baseline that excludes visual information, and impairment scores cannot be reliably predicted. Nevertheless, several findings suggest future promise. With optimized prompting and post-processing, VLMs can classify high-level activities from a few frames, detect motion and grasp with moderate accuracy, and approximate dose counts within 30% of ground truth for mildly impaired and healthy participants, all without task-specific training or finetuning. These results highlight both the current limitations and emerging opportunities of VLMs for data-driven stroke rehabilitation and broader clinical video analysis.

## Author summary

Stroke is a leading cause of long-term disability, with most survivors experiencing upper-limb impairment that limits daily function. Translating rehabilitation advances from animal studies to humans has been hindered by the lack of precise, practical tools to measure movement quantity and quality during therapy. This study explores whether vision-language models (VLMs), cutting-edge AI models trained on vast image-text datasets, can automatically estimate rehabilitation dose and impairment from videos. We evaluated the models on 20 healthy controls and 51 stroke survivors. Our results show that current VLMs lack the fine-grained motion understanding needed for accurate quantification. Nonetheless, these models recognize high-level activities, detect movement and grasp with moderate accuracy, and estimate dose within 30% of ground truth in structured tasks without retraining. We conclude that current VLMs are not yet clinically reliable, but their flexibility and generalization potential make them a promising future tool for data-driven stroke rehabilitation and digital-health applications.

## Introduction

Vision-language models (VLMs) are large-scale deep learning models trained on massive multimodal datasets to jointly interpret visual and textual information. These models have recently achieved remarkable performance across a wide range of computer vision tasks, including image and video captioning, visual question answering, optical character recognition, document understanding, open-vocabulary object detection [1–13], and medical image understanding [14,15]. In this work, we investigate the use of VLMs for analyzing human motion, with a particular emphasis on stroke rehabilitation.

Stroke is the leading cause of disability worldwide. In 2021, there were 93.8 million stroke survivors in the world [16], with upper limb impairment occurring in approximately 50–80% of cases [17]. Most patients remain unable to perform daily activities independently six months post-stroke, reducing quality of life and imposing enormous societal and economic burdens. In animal models of stroke, high numbers of movement repetitions have been shown to improve abnormalities in motion quality [18–20]. Unfortunately, clinical rehabilitation research has largely failed to translate this fundamental work from animals to humans [21–23]. A major barrier is the absence of precise, practical methods for quantifying both training dose (i.e., the number of movements performed during rehabilitation) and impairment level (i.e., the quality of those movements) [24,25].

Previous approaches utilizing machine learning for automatic dose quantification [26–28] and impairment quantification [29,30] from video and wearable-sensor data relied on training datasets with similar characteristics to the test data. This reliance on similar data represents a fundamental limitation for such approaches, given that training data in this domain is very scarce (the largest publicly available dataset contains only 51 subjects [27]). In contrast, VLMs are, in principle, able to directly process images with very different lighting, backgrounds, camera angles and content, as they are trained on enormous amounts of data. In that sense, they hold great promise for digital health applications where video data are highly heterogeneous, such as remote patient monitoring, interactive diagnostic assistance, gait analysis, and surgical training. Recent work has shown the promise of VLMs for clinical gait analysis [31], though performance on fine-grained domain-specific action recognition remains limited [32].

Here, we evaluate the ability of VLMs to perform three rehabilitation tasks: (A) identifying high-level rehabilitation activities, (B) quantifying training dose, and (C) quantifying motor impairment, using a cohort comprising 20 healthy controls and 51 stroke survivors. Activity identification serves as a proof of concept of whether VLMs can recognize what is happening in a rehabilitation video. Dose and impairment quantification—our focus—can be formulated as human-motion recognition problems, where the goal is to identify subtle movements. Our results indicate that current VLMs lack the fine-grained understanding of human motion required for precise quantification. For dose estimation, VLM performance is comparable to a baseline that relies solely on transition statistics between functional primitives, without access to the video data. For impairment assessment, the VLM fails to consistently provide correct responses for any of the items in the Fugl–Meyer assessment [33].

Despite these negative results, several of our findings are moderately encouraging and point to the future potential of VLMs in data-driven stroke rehabilitation and other digital medicine applications. We observe that these models can classify high-level activities from very few frames. They can also identify the presence of motion and grasp with moderate accuracy. For a highly structured rehabilitation task, incorporating carefully engineered postprocessing optimized on a few held-out individuals yields dose estimates within 30% of the ground truth for mildly impaired and healthy subjects. While this falls short of the 10% error threshold below which counting inaccuracies become unlikely to affect recovery outcomes, it represents a promising step toward precise rehabilitation dose quantification.

## Materials and methods

This section first describes the VLMs used in this study, the evaluation cohort, and data acquisition. We then detail our methodology for applying VLMs to three tasks in stroke rehabilitation, detailing our prompting strategies, baselines, and evaluation metrics.

### Ethics statement

All subjects in the videos used in this study provided written informed consent in accordance with the Declaration of Helsinki. The study was approved by the Institutional Review Board at the New York University Grossman School of Medicine.

### Models and cohort

#### Vision-language models (VLMs).

VLMs are powerful multimodal models that can function as video question-answering systems. Their inputs are a set of video frames and a free-form textual question or instruction called a “prompt.” Their output is a free-form textual answer. In simplified terms, a VLM consists of a pre-trained vision encoder connected to a large-language model (LLM) with a vision-to-text connector. The textual and visual inputs to a VLM are first converted to tokens, which are numerical vector representations that serve as the fundamental units for reasoning and generation. The tokens are then fed through the LLM to generate a sequence of output tokens. This sequence is converted to text to produce the final output of the VLM.

VLMs are trained in two stages [34]. In the first stage, the connector learns to align the visual and textual representations using a large dataset of image-text pairs. The second stage fine-tunes the vision encoder, connector, and large-language model jointly to answer questions, describe scenes, and follow instructions about images and videos. In this work, we used 15 state-of-the-art open-source vision-language models of various sizes from 6 model families released in the past two years: LLaVA-NeXT-Video [1], LLaVA-OneVision [2], NVILA [3], Qwen2.5-VL [4], InternVL3 [5], and InternVL3.5 [6]. More details on how inputs are processed for each model family are provided in Section B in S1 Appendix. Our evaluations were done with lmms_eval [35,36], an open-source, standardized evaluation framework.

#### Evaluation cohort and data acquisition.

This study is based on a cohort of 71 individuals presented in Table 1, consisting of 20 healthy subjects and 51 stroke patients. The stroke patients are separated into three motor impairment levels—mild, moderate, severe—based on the Fugl-Meyer assessment (FMA), a standardized 33-item clinical scale used to quantify motor impairment of the arm, wrist, and hand after stroke [33]. The subjects were recorded in an inpatient rehabilitation gym under two different contexts: (1) rehabilitation based on activities of daily living (ADL) and (2) impairment quantification via the FMA. The recordings were captured simultaneously from two different camera angles, using two high-definition cameras (Ninox, Noraxon) placed orthogonally less than 2 m from the subject, at a resolution of 1088 x 704 pixels and 60 or 100 frames per second.

**Table 1:** Demographic and clinical characteristics of the cohort. Mean (range) is reported where applicable. ADL was evaluated on the full cohort, while FMA was evaluated on a subset of 4 healthy and 24 stroke subjects (see Table 2).

Characteristic	Healthy	Stroke
# of subjects	20	51
# of ADL trials	917	2002
# of FMA trials	83	524
Age	62.5 (42.0−82.9) years	57.7 (21.2−84.2) years
Sex	9 Female, 11 Male	28 Female, 23 Male
Paretic Side	NA	28 Left, 23 Right
Fugl–Meyer Score	66	43.3 (8−65)
Impairment Level	NA	20 Mild, 23 Moderate, 8 Severe
Time Since Stroke	NA	5.5 (0.3−38.4) years

The ADL videos include nine activities: brushing teeth, combing hair, applying deodorant, drinking water, washing face, eating, putting on/taking off glasses, and performing repetitive target-directed movements—moving a toilet paper roll horizontally on a table (radial tabletop task, RTT) and vertically on a transparent shelf with different shelf heights (shelf) (see Section C in S1 Appendix for a more detailed description). Each subject performed each activity for 3−5 trials.

In the FMA videos, subjects performed one of 33 movement items under the supervision of a trained expert. Each item is scored from 0 to 2. The aggregate FMA score is a number between 0 (maximally impaired) and 66 (healthy). A more detailed description of the FMA is provided in Section J in S1 Appendix.

### VLMs for stroke rehabilitation

**Fig 1:**
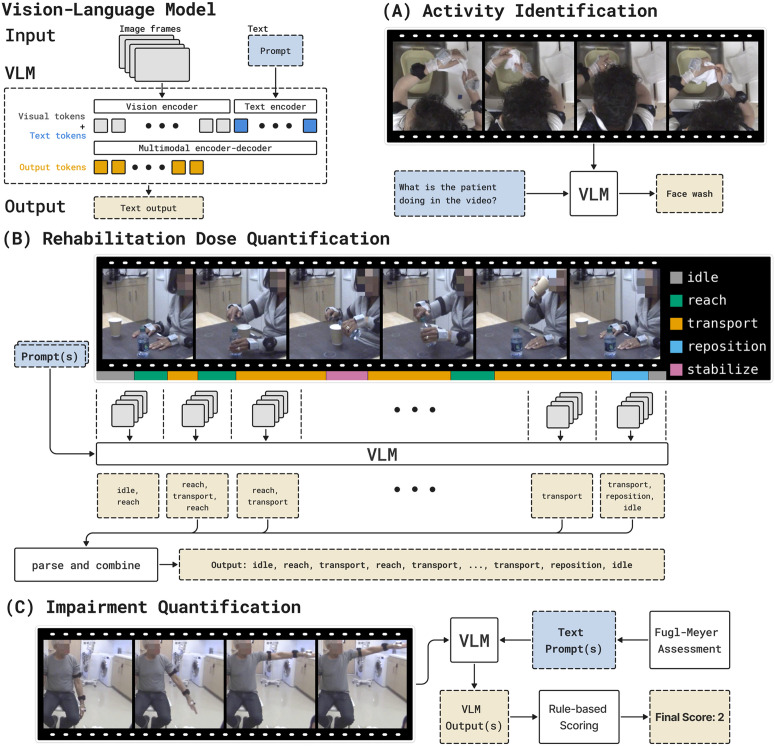
Vision-language models (VLMs) for data-driven stroke rehabilitation. **(Top-Left)** A VLM can function as a video question-answering system. The question, or *prompt*, and image frames from the video are separately encoded into tokens, which are fed-forward through a transformer-based backbone. The backbone’s output is then decoded into text. **(A)** *Activity identification:* The VLM is provided 8 frames uniformly sampled from a video (4 frames are shown due to space constraints) and a description of nine rehabilitation activities. The VLM output classifies the activity in the video. **(B)** *Dose quantification:* The VLM is provided a video segment along with a textual prompt. Its output is then utilized to classify the segment into one of five functional motions or primitives. Rehabilitation dose is quantified by counting the primitives over the whole video. **(C)** *Impairment quantification:* The VLM processes video segments of subjects performing mobility exercises from the Fugl-Meyer Assessment (FMA), a standard clinical evaluation of impairment. The input prompt is the corresponding FMA item. The outputs are aggregated to estimate the FMA score of the subject.

Fig 1 shows how we propose to apply VLMs for dose and impairment quantification from videos, and also for identification of high-level rehabilitation activities. Specialized prompts are provided to the model, along with multiple video frames, in order to solicit the information required for each task. In the case of dose quantification, the video is divided into short segments, and the VLM is asked to classify each segment into one of five basic functional movements or *primitives*, which are then counted to determine the rehabilitation dose [37]. In the case of impairment quantification, each prompt is based on an item from the Fugl-Meyer assessment (FMA). The model outputs are then combined to produce an estimate of the FMA score.

We evaluate VLMs on three stroke rehabilitation tasks: (A) activity identification, (B) dose quantification, and (C) impairment quantification. Table 2 summarizes the subject splits for each task. For some tasks, we reserved a small set of held-out subjects for prompt optimization (PO) and tuned prompts on these subjects before evaluating on the independent test cohort (TC); for others, we applied VLMs directly without any finetuning or prompt optimization. We include the rationale for our dataset splits in Section A in S1 Appendix.

**Table 2:** Task-specific held-out and test cohorts used for VLM evaluation. TC = Test cohort; PO = Held-out subjects used for prompt optimization.

Task	Impairment Level (Control, Mild, Moderate, Severe)
(A) Activity Identification	TC: 18, 20, 23, 8; PO: 2, 0, 0, 0
(B) Dose Quantification	
B.1 General (all activities)	TC: 5, 3, 2, 0
B.2 RTT/Shelf (optimized)	TC: 18, 20, 23, 7; PO: 2, 0, 0, 1
(C) Impairment Quantification	TC: 4, 10, 10, 4

#### Activity identification.

As shown in Fig 1(A), we formulated activity identification as a classification task, where the classes are nine rehabilitation activities. We provided the VLM with two inputs: eight frames, uniformly sampled from a rehabilitation video, and a classification prompt that describes the nine activities and asks the model to identify which one is depicted in the video frames. The textual output of the VLM indicates the estimated activity.

We considered two alternative strategies for producing activity descriptions in the classification prompt. The first strategy was *direct prompting*, where we generated one-sentence descriptions of the activities using ChatGPT 5 (https://chat.openai.com) by providing screenshots of the supplementary material in [27]. The second strategy was *optimized prompting*, specifically adapted to the Qwen2.5-VL-7B-Instruct model. We first asked the VLM for a description of the objects in the work station for held-out videos from two control subjects. We then modified the activity descriptions to match the wording of the model. For example, “comb” became “small rectangular object.” Surprisingly, small changes like this boosted the accuracy from 53.4% to 77.5%. We include transcripts of both types of prompts in Section E in S1 Appendix.

#### Dose quantification.

We formulated the problem of measuring rehabilitation dosage as a sequence estimation task, where the goal is to identify the sequence of fine-grained motions carried out by a subject during a video. The five motions of interest, called *functional primitives* (see Table 3), are *reach* (move to contact a target object), *reposition* (move proximate to a target object, e.g., the initial neutral spot), *transport* (move a grasped target object), *stabilize* (hold a target object still), and *idle* (stand at the ready near a target object).

**Table 3:** Definition and motion-grasp decomposition of functional primitives for dose quantification. The table provides definitions of the five functional primitives [37], and shows how answers to the questions “Is the hand moving significantly?” and “Is the hand grasping an object?” can be used to identify a primitive (except for reach and reposition, which must be distinguished based on terminal grasp).

Primitive	Definition	Motion	Grasp	Grasp at end
Reach	Move into contact with a target object	Present	No	Yes
Transport	Convey a target object	Present	Yes	Either
Reposition	Move without the purpose of future contact	Present	No	No
Stabilize	Keep a target object still	Minimal	Yes	Either
Idle	Stand at the ready	Minimal	No	Either

Fig 1(B) illustrates the high-level approach for primitive sequence estimation. We first divided the video into segments with duration 0.533 s. We constrained our procedure to produce one primitive per segment, as initial experiments allowing multiple primitives led to overcounting. The VLM was queried one or more times per segment—with each query receiving eight uniformly sampled frames and a hand-designed prompt—and its outputs were aggregated into a single per-segment primitive (e.g., *transport*). These per-segment primitives were combined to form the final sequence, with identical consecutive primitives collapsed (e.g., *idle*-*transport*-*transport*-*reach* becomes *idle*-*transport*-*reach*).

More specifically, we employed a *Decomposed Prompting* strategy, which queries the VLM twice per segment—one querying whether the hand is moving significantly, and one querying whether the hand is grasping an object. As shown in Table 3, the combination of these two binary outputs uniquely identifies three of the five primitives. The remaining ambiguity is between *reach* and *reposition*, which both involve motion without a grasp. These were resolved with a terminal-grasp heuristic: if the VLM predicts either *transport* or *stabilize* within two seconds of the primitive’s start time, we classified it as *reach*; otherwise, it was classified as *reposition.* The 2-second threshold was validated on 21 videos from two control subjects outside the evaluation set. In Section H in S1 Appendix, we compare the *Decomposed Prompting* strategy to one that directly asks the VLM to output the primitive and include ablations on alternative segment durations and frame sampling rates. For context, we refer the reader to Fig A in S1 Appendix for statistics describing the durations of primitives within the videos.

Two baselines provide context for our results. The *Omniscient* baseline is an oracle analog of *Decomposed Prompting*: rather than querying the VLM, the motion and grasp binary answers are set to ground-truth values derived from the primitive at the center frame of each segment. It represents the best possible performance achievable by any model under the one-primitive-per-segment constraint and the 2-second *reach*/*reposition* heuristic. The *Markov* baseline only relies on first-order transition statistics, estimated from the ground-truth primitive sequence. Two 2×2 transition matrices (one for motion, one for grasp) were estimated from the test set of B.1 in Table 2; predictions were then generated by starting in the no-motion/no-grasp state and randomly sampling subsequent transitions until the end of the video. This baseline merely exploits the likelihood of different transitions between primitives, but does not have access to the video.

#### Optimizations for dose quantification.

Here, we describe modifications to the *Decomposed Prompting* strategy. We evaluated these optimizations using Qwen2.5-VL-32B-Instruct on B.2 in Table 2.

- **Contextual prompting:** In an attempt to reduce over-segmentation (see Fig 4(c)), we incorporated the VLM’s predictions from the previous segment directly into the text prompt for the current segment (see Section G in S1 Appendix for the prompts).
- **Cropping:** Since most primitives can be identified by observing the hand in the upper extremity of interest, we employed a 2D pose model [38] to localize and crop the hand region, producing cropped images that were provided as visual input to the VLM. First, we prompted the VLM to identify all individuals in the initial video frame and designated the person with the largest bounding box as the subject. We then used a 2D pose model to obtain COCO keypoints [39] for the subject. We selected the elbow and wrist keypoints and estimated the hand region by extending the elbow-to-wrist vector by a factor of 0.7. If, for any frame in a segment, the lowest confidence score among the elbow and wrist keypoints was below 0.9, we abstained from cropping and passed on the original frames to the VLM. If quick movement was detected, we extracted a moving crop that linearly interpolated the hand position across the segment; otherwise, we extracted a still crop centered at the hand’s average position over the segment. Quick movement was detected when the average pixel movement in the horizontal or vertical direction exceeded 15. We ensured that crops respected image boundaries. The crop size was 224*x*224 pixels.
- **Pose-Refined promptIng Module (PRIM-RS):** We propose PRIM-RS as a pipeline optimized for dose quantification for the RTT and shelf tasks (B.2 in Table 2). This system, which was tuned on a held-out set of videos from two control subjects and one severe patient, operates on 0.267-s segments and employs specialized binary prompts, a pose-informed decision pathway, and carefully designed post-processing. This small PO set could raise concerns about potential bias toward a limited set of movement characteristics. However, optimization served to expose VLM failure modes and inform task-level design choices—not to fit subject-specific motion patterns—and the resulting prompts make no reference to the subjects’ movements (see Section G in S1 Appendix). We include full details in Section D in S1 Appendix.

#### Metrics for assessing dose quantification.

For evaluation, we have access to per-frame ground-truth primitive labels. These annotations pertain to a target side (left or right) and were meticulously labeled by trained annotators with Cohen’s kappa ≥0.96 between the labelers and the expert. We obtain the predicted sequence for the target side and compare the predicted and ground-truth sequences using three metrics: *edit score*, *action error rate*, and *relative counting error.*

Denote the predicted sequence by *P* and the ground-truth sequence by *G*. The *edit score* ES(*G*,*P*) and the *action error rate* AER(*G*,*P*) [27] are normalized versions of the *Levenshtein edit distance* L(*G*,*P*), which counts the minimum number of operations needed to transform one sequence into the other. The operations are insertion, deletion, and substitution. For example, *idle-reach-transport* has an edit distance of 2 from *reach-stabilize-transport* (replace *idle* with *reach* and *reach* with *stabilize*, or delete *idle* and add *stabilize*).


$$\begin{array}{c} \text{ES}(G,P)=\left(1-\frac{L(G,P)}{\max(\text{len}(G),\text{len}(P))}\right)\times 100\;\text{AER}(G,P)=\frac{L(G,P)}{\text{len}(G)}, \end{array}$$

where len indicates the length of a sequence.

Note that a higher ES is better, while a lower AER is better. For a null prediction, the ES is 0 and the AER is 1. We report both metrics because the ES is common in the action recognition literature [27,40–43] and the AER is better suited to evaluating estimation of highly granular motions like functional primitives, as it more heavily penalizes long incorrectly estimated sequences [27].

The final metric is the *relative counting error* RCE(*G*,*P*), which directly evaluates rehabilitation dose quantification based on primitive counts. The RCE aggregates the counting errors across the primitives and normalizes the sum by the ground-truth sequence length:


$$\begin{array}{c} \text{RCE}(G,P)=\frac{1}{\text{len}(G)}\sum_{p\in 𝒫}|c(p,G)-c(p,P)|, \end{array}$$

where 𝒫 denotes the set containing the five primitives and *c*(*p*,*S*) the number of times primitive *p* occurs in the sequence *S*.

#### Impairment quantification.

As shown in Fig 1(C), we leveraged the Fugl-Meyer Assessment (FMA) to perform impairment quantification using VLMs. The FMA consists of multiple items designed to evaluate the movement quality of a subject. We used the VLM to rate each item by providing (1) a short clip of a subject performing an FMA item and (2) the rating instructions given to a trained expert for that item. The textual output of the VLM was then used to estimate the score for the item: 0 (worst), 1, or 2 (best).

**Fig 2:**
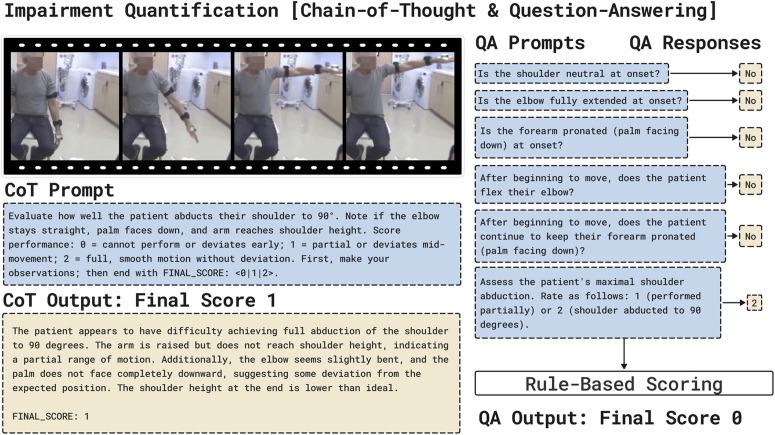
Strategies for Applying VLMs to Impairment Quantification. To assess impairment using VLMs, we evaluated two approaches. Chain-of-Thought supplies the VLM with all relevant contextual information in the prompt, requesting the model to reason through its observations and conclude with a final score. Rule-based question-answering prompts the VLM with a list of binary questions. The answers are then aggregated to deduce a final score. Here, the first “No” set the final score to “0” (the rest of the answers do not matter, but are shown for illustration). For this specific video, both methods ended up with an incorrect score: the ground-truth score was 2.

We employed two general strategies for prompting, explained in Fig 2. For *Chain-of-Thought (CoT)* prompting [44], we provided the VLM with all relevant rating information in the prompt and asked it to reason through its observations and conclude with a final score. For *Rule-based Question-Answering (QA),* we designed a list of questions tailored to each FMA item. Each question elicited a binary response. Depending on the response, a score was assigned or the next question was presented. This process continued until the final question, which forced a final rating. See Section K in S1 Appendix for the specific CoT and QA FMA prompts.

Since the subject performed each FMA item three times, we selected the middle repetition for the VLM to rate, using the start and end times of this middle repetition to define clip boundaries. Each subject was filmed using two camera angles throughout the FMA session: one positioned directly in front of the subject and another facing their paretic side (see Section J in S1 Appendix). We chose the camera angle best suited for rating a particular question.

For most FMA items, we uniformly selected eight frames from the clip. This low number should be sufficient for accurate rating because the sampling rate captures the relevant motion characteristics and the clips are short (95% have duration <10 s; see Section J in S1 Appendix). An exception is the FMA section named *Coordination/Speed*, where subjects are instructed to rapidly move their finger between their nose and knee five times to test for tremor, dysmetria, and movement speed. For tremor and dysmetria, we segmented the video using eight frames per 0.267-s segment, rated each segment, and averaged the rating across segments to generate a final rating. For movement speed, we similarly segmented the video and prompted the VLM to count the number of “touches” detected on the nose and knee within each segment. We then identified the minimum time point at which the cumulative touch counts for both knee and nose exceeded five. The final score was then determined by comparing this minimum time point between the paretic and healthy sides.

## Results

### VLMs accurately classify high-level activities

As a proof of concept of the ability of VLMs to understand the semantic content in rehabilitation videos, we consider the problem of identifying rehabilitation activities, as described under “Activity identification” in the Methods. Our evaluation set comprises 640 videos featuring 18 healthy subjects and 51 stroke patients.

**Fig 3:**
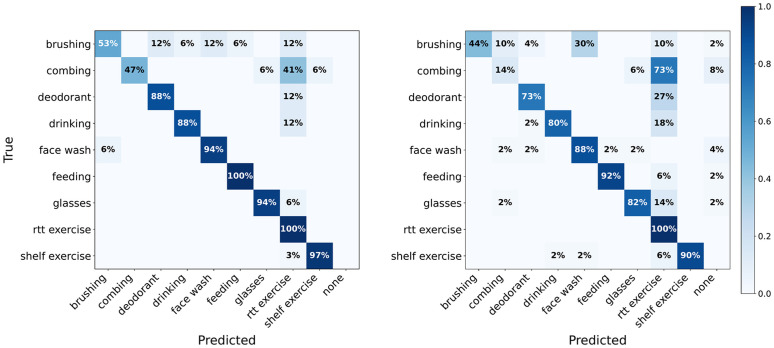
VLMs accurately classify high-level activities, with lower accuracy for stroke patients. Confusion matrices for activity identification with Qwen2.5-VL-7B-Instruct using optimized prompts (see Methods). Each cell shows the fraction of samples from a true activity (row) that were predicted as a given activity (column). **Left:** Healthy controls (*N* = 188 videos, 18 subjects, accuracy 87.2%). **Right:** Stroke patients (*N* = 452 videos, 51 subjects, accuracy 73.5%). Accuracy is notably lower for stroke patients, with increased confusion between related activities. See Fig B in S1 Appendix for a comparison between direct and optimized prompting.

Fig 3 shows confusion matrices for optimized prompting (see Methods) separately for healthy controls (left) and stroke patients (right). Optimized prompting achieves an overall average accuracy of 77.5%: 87.2% for controls and 73.5% for stroke patients. Errors are concentrated in the face-wash/brushing and combing/RTT pairs, likely due to the small sizes of the objects involved. For stroke patients, these confusions are more pronounced and additional errors emerge across activities. Interestingly, the optimized prompt substantially boosts the performance of Qwen2.5-VL-7B-Instruct, but not that of the larger models in the Qwen family (56.4% for Qwen2.5-VL-32B-Instruct and 64.7% for Qwen2.5-VL-72B-Instruct), illustrating that prompt optimization is specific to a given model.

Stratifying stroke patients by severity reveals a monotonic decrease in accuracy: 80.0% for mild (*N* = 180, 20 subjects), 73.2% for moderate (*N* = 205, 23 subjects), and 56.7% for severe (*N* = 67, 8 subjects). This trend suggests that both environmental context (workstation layout, objects, scene composition) and motion quality contribute to performance. Qualitatively, we observed that more severely impaired subjects sometimes perform the activity in an atypical or incomplete way that is difficult to recognize visually. This impacts model performance because our evaluation samples 8 frames uniformly from the video, and likely the control subjects achieve a recognizable pose in at least one of those frames, while severely impaired subjects may not. In conclusion, while accuracy drops with stroke severity, VLMs can still classify activities with relatively high accuracy from a small number of frames, provided the prompts are optimized on a small held-out set.

### VLMs are able to detect subtle motions to some extent, but not enough for precise dose quantification

We evaluated the *Decomposed Prompting* strategy on a test set of 90 videos comprising nine activities for each of five control subjects and five mild/moderate stroke patients (see “B.1 General (all activities)” in Table 2). The total number of primitives in the test dataset was 4,059. The evaluation was fully independent, without any prompt optimization.

Table 4 shows the results of applying 15 different VLMs for dose quantification using the proposed framework. The relative counting error is 65% or greater for all models. In fact, the best models achieve only slightly better performance than the Markov baseline, which does not take into account the video content. Using one of the best models, Qwen2.5-VL-32B-Instruct, we evaluated the effect of visual curation (cropping the input frames around the relevant upper extremity) and contextual prompting (see Methods for more details). Cropping boosts performance slightly, while contextual prompting does not.

**Table 4:** In dose quantification, VLMs perform only slightly better than a baseline that is independent from the visual input. The table shows the edit score (ES), action error rate (AER), and relative counting error (RCE) of 15 VLMs with different sizes. Arrows indicate the direction of better performance. Green indicates the best result (including ties) among all models, and blue the second-best result. Cells show mean ± 1 sem. The best models are barely better than the Markov baseline, which only relies on transition statistics, and are very far from the Omniscient baseline, an upper bound on performance based on the ground-truth primitive sequences. The bottom of the table reports the performance of Qwen2.5-VL-32B-Instruct with engineered inputs. Evaluation was completely independent, without any prompt optimization.

Model	Edit Score ↑	Action Error Rate ↓	Rel. Counting Error ↓
**Baselines**			
Markov (does not rely on videos)	46.32 ± 0.91	0.71 ± 0.08	0.63 ± 0.08
Omniscient	88.47 ± 0.87	0.12 ± 0.01	0.11 ± 0.01
InternVL3-78B	**49.83 ± 1.21**	0.68 ± 0.09	0.73 ± 0.09
InternVL3.5-2B	34.20 ± 1.98	0.81 ± 0.12	0.80 ± 0.12
InternVL3.5-8B	8.17 ± 1.24	0.92 ± 0.01	0.93 ± 0.01
InternVL3.5-38B	40.20 ± 1.49	0.68 ± 0.03	0.80 ± 0.03
InternVL3.5-30B-A3B	25.54 ± 1.94	0.78 ± 0.02	0.87 ± 0.03
LLaVA-NeXT-Video-7B	46.18 ± 1.76	**0.65 ± 0.07**	**0.68 ± 0.07**
LLaVA-NeXT-Video-72B	23.08 ± 1.65	0.78 ± 0.02	0.97 ± 0.03
LLaVA-OneVision-0.5B	20.97 ± 1.44	0.80 ± 0.01	0.96 ± 0.02
LLaVA-OneVision-7B	42.07 ± 1.31	0.69 ± 0.04	0.78 ± 0.05
LLaVA-OneVision-72B	38.52 ± 1.93	0.72 ± 0.07	0.76 ± 0.08
NVILA-8B	36.98 ± 2.09	0.75 ± 0.09	0.76 ± 0.09
NVILA-15B	24.92 ± 1.21	0.79 ± 0.04	0.95 ± 0.04
Qwen2.5-VL-7B-Instruct	34.75 ± 1.73	0.69 ± 0.02	0.77 ± 0.02
Qwen2.5-VL-32B-Instruct	**46.69 ± 1.62**	**0.65 ± 0.06**	**0.65 ± 0.06**
Qwen2.5-VL-72B-Instruct	44.88 ± 1.58	**0.58 ± 0.02**	**0.65 ± 0.02**
**Engineered inputs**			
(Qwen2.5-VL-32B-Instruct)			
Cropping	48.28 ± 1.53	0.61 ± 0.04	0.62 ± 0.04
Contextual Prompting	48.32 ± 1.61	0.73 ± 0.08	0.83 ± 0.08
Cropping & Contextual Prompting	53.18 ± 1.50	0.70 ± 0.05	0.76 ± 0.05

**Fig 4:**
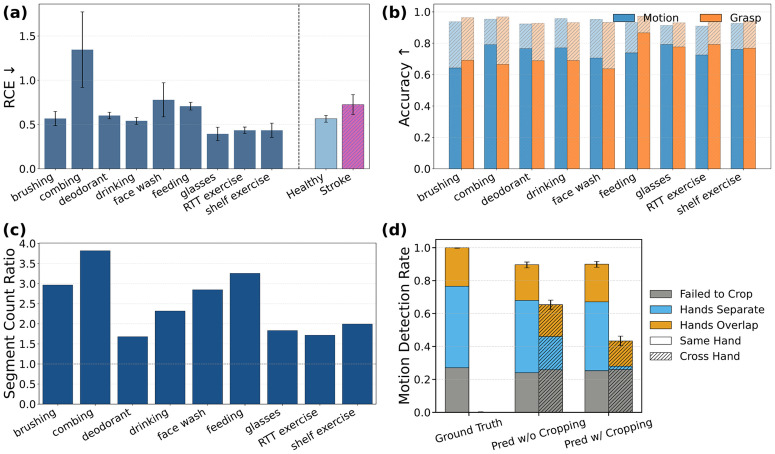
VLMs detect motion and grasp with relatively high accuracy (albeit insufficient for precise dose quantification), but confuse left and right. The graphs provide a nuanced analysis of one of the best-performing models: Qwen2.5-VL-32B-Instruct with cropping. Evaluation was completely independent, with no prompt optimization. **(a)** The relative counting error (RCE ↓) is high (≈50% for most activities) and is worst for *combing*. Healthy subjects show slightly lower RCEs compared to stroke patients. The bars represent mean ± 1 sem. **(b)** The accuracy of the predicted motion and grasp (solid bars) is between 60 and 80% across all activities, indicating moderately accurate detection. The lighter background bars illustrate the performance of the *Omniscient* baseline. **(c)** The model switches predictions between consecutive segments too frequently—especially for combing. **(d)** We isolated segments where only one hand was active. When instructed to track a particular hand, a VLM should detect motion 100% of the time for the moving hand and 0% for the still hand. Empirically, the VLM mistakenly classifies the still hand as moving in >60% of cases, presumably due to the motion in the other hand. Cropping partially alleviates this issue, but can fail and does not help when the hands overlap. The error bars show 95% binomial proportion confidence intervals.

Fig 4 provides a more detailed analysis of one of the best-performing models: Qwen2.5-VL-32B-Instruct with cropping. Fig 4(a) shows that the *relative counting error* (RCE) is close to 50% across activities, with extremely poor performance for combing. The aggregated RCE is lower for healthy subjects than for stroke patients, but is still above 50%. Fig 4(b) reports the performance of the VLM (solid bars) compared to the *Omniscient* baseline (lighter background bars) for motion and grasp detection *at the frame level.* While the predictions are moderately accurate for most activities, the accuracy is not enough to result in precise primitive counts.

Fig 4(c) shows that the VLM primitive estimates are quite unstable, constantly switching between primitives, which results in over-segmentation: 1.5 to 4 times more segments are predicted compared to the number of ground-truth segments after de-duplication.

An interesting limitation of VLMs is that they fail to follow instructions that require differentiating between the left and right hand, despite careful prompt design (see Section G in S1 Appendix). In Fig 4(d), we report the results of an experiment based on segments where one hand is actively moving and the other is not. We prompted the VLM once for each hand, asking whether that hand was currently moving. Ideally, a model should detect motion 100% of the time for the active hand and 0% of the time for the non-active hand. However, we found that the model declared the non-active hand to be *moving more than 60% of the time*. Cropping partially mitigates this issue, but fails for some videos and remains ineffective when the hands overlap. More details on this experiment are provided in Section I in S1 Appendix. We further provide primitive-level results in Section H in S1 Appendix.

### With prompt optimization and post-processing, VLMs can quantify rehabilitation dosage for structured activities to some extent

The results in Table 5 demonstrate that activity-specific optimizations boost performance. Most notably, the post-processing step in PRIM-RS, which involves smoothing to prevent over-segmentation, is critical. Cropping around the desired hand yields additional gains.

**Table 5:** Prompt optimization, cropping and post-processing improve dose quantification. Results of *PRIM-RS* (Pose-Refined promptIng Module—RTT/shelf), which incorporates optimized prompting, cropping and post-processing, compared to *Decomposed Prompting* (based on non-optimized predefined prompts). Evaluation is performed on 132 videos from all available subjects outside the prompt-optimization set, including each RTT or shelf task video a subject completed. *PRIM-RS* operates on 0.267-s segments, half the 0.533-s default of *Decomposed Prompting.* We use the aerial camera angle for the shelf task and the side view closer to the hand of interest for the RTT task. PRIM-RS improves upon Decomposed Prompting for the three metrics. Cropping around the hand of interest and careful post-processing provide gains in performance. Arrows indicate the direction of better performance. Cells show mean ± 1 sem.

Prompt Method	Edit Score ↑	Action Error Rate ↓	Rel. Counting Error ↓
PRIM-RS	66.85 ± 1.44	0.40 ± 0.02	0.40 ± 0.03
– w/o cropping	64.41 ± 1.54	0.46 ± 0.03	0.46 ± 0.03
– w/o post-processing	51.72 ± 1.22	0.74 ± 0.04	0.74 ± 0.05
– w/o cropping/post-processing	47.17 ± 1.11	0.90 ± 0.09	0.95 ± 0.10
Decomposed Prompting (0.267-s)	48.85 ± 1.15	0.93 ± 0.14	1.00 ± 0.15
Decomposed Prompting (0.533-s)	56.34 ± 1.08	0.74 ± 0.24	0.75 ± 0.24

**Fig 5:**
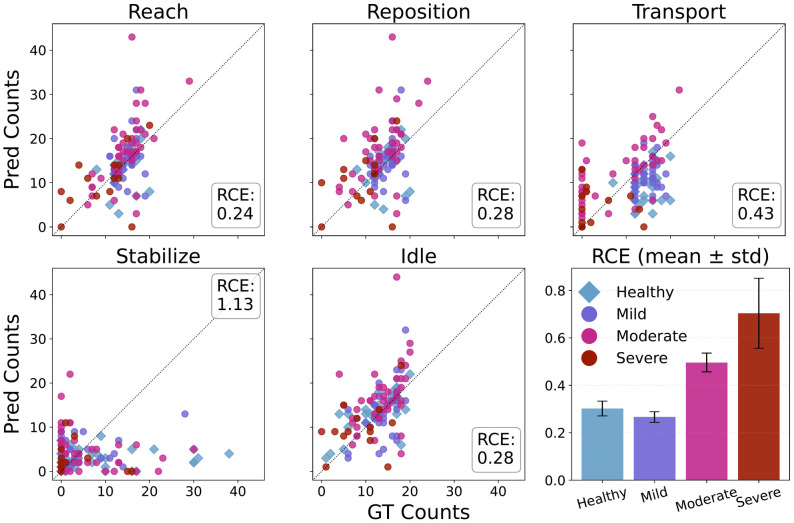
With prompt optimization and post-processing, VLMs can quantify rehabilitation dosage for structured activities to some extent. The first five panels show scatterplots comparing the predicted and ground-truth counts for 132 rehabilitation videos corresponding to two structured tasks (radial table top and shelf). Predictions are obtained using the proposed PRIM-RS method, which provides optimized prompts to the VLM Qwen2.5-VL-32B-Instruct and applies post-processing to its output. The relative counting error (RCE) is primitive-specific, measuring the counting error for each primitive normalized by its total number of ground-truth instances in the dataset. The RCE is moderately low at ≈25% for *reach*, *reposition*, and *idle*. It is subpar for *transport* and *stabilize*, illustrating the difficulty of differentiating these primitives. The bottom-right plot shows the average RCE across subjects with different impairment levels: the performance degrades as the severity level increases.

Fig 5 displays the dose quantification results for PRIM-RS. For the *reach*, *reposition*, and *idle* primitives, the *per-primitive counting error* (see caption) is moderately accurate at approximately 25%. This mainly stems from differences in movement speed across severity levels, and from the fact that *stabilize* durations can be extremely brief. The bottom right of Fig 5 shows the mean performance for subjects with different impairment levels. PRIM-RS performs considerably better (25–30%) for healthy subjects and mild patients, but its performance deteriorates for moderate and severe patients, whose movements are irregular and frequently idiosyncratic.

### VLMs are not able to perform impairment quantification

We evaluated the proposed framework on 899 videos from a cohort of 28 subjects (see “(C) Impairment Quantification” in Table 2). Evaluation was completely independent, with no prompt optimization. We applied the *Chain-of-Thought (CoT)* and *Rule-based Question-Answering (QA)* prompting strategies described in the Methods section.

**Fig 6:**
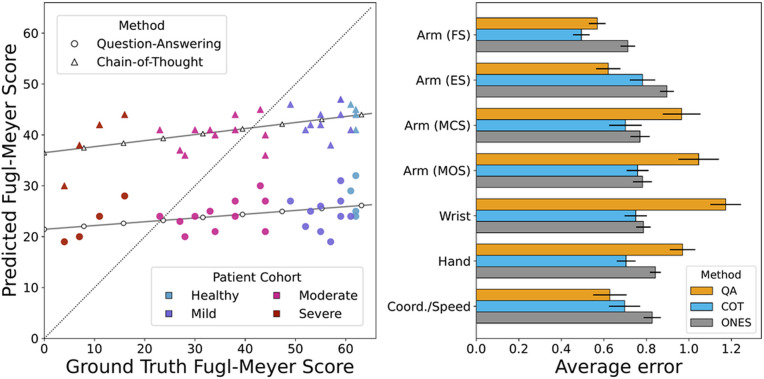
VLMs fail at impairment quantification. **Left:** The scatterplot shows the ground-truth Fugl-Meyer score assessed by a trained human expert against the predicted Fugl-Meyer score by the VLM Qwen2.5-VL-72B-Instruct. The points would lie along the dotted diagonal line for a model that matches human rating. Instead, for two different prompting methods, the predicted Fugl-Meyer score is nearly constant across severity levels. **Right:** The bar plots show the average error of the VLM for different subsections of the Fugl-Meyer assessment, again using two prompting methods. For comparison, ONES displays the average error for a model that only predicts 1. The subsections are, from top to bottom, Arm—Flexor Synergy, Arm—Extensor Synergy, Arm–Movement Combining Synergy, Arm—Movement Out of Synergy, Wrist, Hand, and Coordination/Speed. Both methods perform comparatively to the non-informative ONES baseline. Bars show mean ± 1 sem.

Fig 6 shows the results for Qwen2.5-VL-72B-Instruct. The predicted Fugl-Meyer score in the left plot is essentially constant throughout the spectrum of severity levels, indicating that the VLM fails to quantify impairment. The right plot breaks down the average error for all patients and scoring items for different subsections of the FMA. Errors for the two methods are similar to a non-informative model that ignores the visual input and returns a score of 1.

## Discussion

This study reaches a nuanced conclusion regarding the current capabilities of vision-language models (VLMs) for human motion understanding.

On the positive side, after prompt optimization on a limited number of held-out subjects, VLMs identify high-level activities accurately, and can be engineered to perform fine-grained motion identification in highly structured contexts. Moreover, VLMs can detect the presence of substantial motion or grasp with moderate accuracy, even without prompt optimization. Remarkably, these capabilities emerge without any task-specific training or finetuning on similar data, distinguishing VLMs from traditional machine-learning approaches that rely heavily on domain-specific supervision. This is particularly attractive for digital medicine applications, such as stroke rehabilitation, where annotated data is scarce.

**Fig 7:**
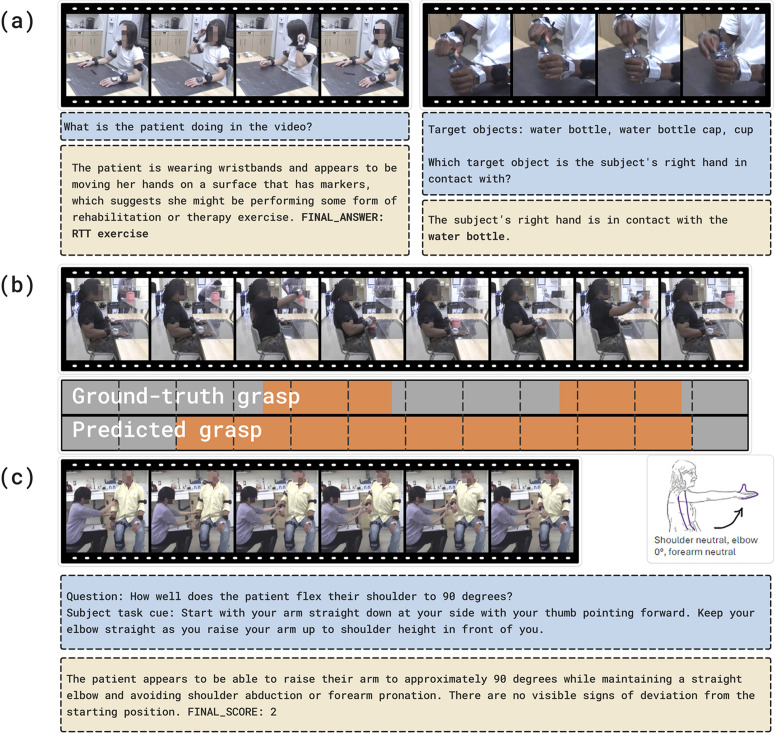
Failure modes of VLMs applied to stroke rehabilitation. **(a) Large object bias:** Left: The model misclassifies a combing activity as an RTT exercise, likely driven by contextual cues from larger objects such as the black mat and wristbands. Right: The model exhibits hand attribution errors, potentially due to the left hand’s interaction with a dominant object (water bottle). **(b) Overreliance on 2D semantics:** Timeline of a 6 s video with dotted lines marking 0.533 s segments. Colors indicate grasp state: gray (no grasp), orange (grasp), and black (mixed). The ground truth contains two distinct grasps. When queried, “Is the subject’s right hand grasping the pink object? Answer ‘Yes’ or ‘No’ directly.” for each segment, the VLM misinterprets visual proximity as physical contact and fails to distinguish the two separate grasp events. **(c) Hallucination:** A patient with severe impairment attempts a shoulder flexion task (ground truth Fugl-Meyer score of 0). For reference, the diagram on the right depicts successful completion of the task. The model hallucinates movement and incorrectly reports task success.

On the negative side, VLMs are unable to capture the subtle kinematic details required for precise quantification of rehabilitation dose or motor impairment, even after careful engineering. This is supported by our quantitative results, and also by qualitative observations that reveal several critical failure modes (see Fig 7):

- *Object bias:* VLMs tend to focus on large objects; for instance, several combing videos were misclassified as RTT seemingly because the presence of a black mat led the model to infer “some form of exercise or therapy.”
- *Limited sensitivity to fine movements:* The models fail to detect wrist motions like twisting and rotating, leading to poor performance on activities such as face washing or feeding.
- *Overreliance on 2D semantics:* VLMs may infer a grasp when a hand merely hovers near an object, lacking the depth reasoning needed to recognize actual contact.
- *Hallucinations:* In some cases, the models fabricate motion, reporting that a severely impaired patient performs a task correctly when barely any movement occurs.

In addition, we identify a key methodological limitation: VLM architectures require many tokens to represent a single frame. This results in a tradeoff due to computational constraints: either we reduce the sampling FPS or shorten the temporal context. The former is problematic because it intrinsically fails to capture fine-grained actions. The latter is problematic because, without temporal context about the movement speed of a patient, the VLM struggles to distinguish between the *transport* and *stabilize* primitives, as these depend strongly on the individual’s pace. It also results in oversegmentation: motion blur, occlusion, and camouflage (when the object boundary melds with the immediate background) are common in hand–object interactions, so objects may intermittently appear or disappear from view, causing the VLM to rapidly alternate its predictions for grasp detection. Attempts at contextual prompting (i.e., embedding prior information in the textual input to the VLMs) proved ineffective. Because of this inherent bottleneck in how VLMs represent video, fine-tuning on specialized datasets may not resolve their fundamental inability to capture fine-grained motion.

These limitations align with broader findings from the computer vision community. ActionAtlas [32], a benchmark for domain-specialized action recognition, finds that state-of-the-art VLMs perform well below human baselines, with visual hallucinations as the dominant failure mode—consistent with our observations. Similarly, MotionBench [45], a benchmark for fine-grained motion comprehension, finds that existing VLMs perform poorly across a range of motion-oriented tasks, with repetition counting being particularly challenging. These difficulties are not confined to VLMs: even specialized video architectures fall far short of human performance on fine-grained skill assessment [46].

Our findings suggest that achieving the level of human motion understanding required for data-driven stroke rehabilitation and other video-based digital health applications will necessitate both new datasets and methodological advances. On the data side, new large-scale datasets for training [47–49] and benchmarks for evaluation [32,50–52] are emerging. Most existing benchmarks still target high-level semantic understanding, but a small set now demands the temporal and spatial precision needed for fine-grained motion analysis [32,51]. A significant bottleneck nevertheless persists for digital health: the lack of large-scale, multi-site data with annotations of the granularity required for analyzing subtle human motion. Our own dataset reflects this gap: it was collected at a single clinical site, in a single inpatient rehabilitation gym, under controlled lighting, and with standardized camera placement. Broadening to multi-site recordings with greater environmental variation is an important direction for future work.

On the methodological side, overcoming the VLM failure modes identified in this study will require progress in efficient video encoding [53], accurate detection of small-scale objects and subtle movements [54], monocular 3D understanding [55], and long-term spatial memory for occluded objects [56]. More broadly, one promising direction involves rethinking VLM architectures to incorporate spatially and temporally precise representations of body and object movement, such as parametric body models [57,58] and fine-grained motion annotations [27,59]. Ultimately, the goal is to achieve clinical-grade kinematic precision without sacrificing the broad language accessibility that makes these systems useful in practice.

## Supporting information

S1 Appendix Supplementary methods and results. Supporting Information for the main text: choice of dataset split (Section A); VLM input preprocessing (Section B); more details on the activity videos (Section C); the PRIM-RS method (Section D); prompts for activity identification (Section E); direct vs. optimized prompting for activity identification (Section F); prompts for dose quantification (Section G); further results for dose quantification (Section H); the Fig 4(d) experimental procedure (Section I); more details on the Fugl-Meyer assessment videos (Section J); and prompts for impairment quantification (Section K).
(PDF)

S1 Data Rule-based question-answering prompts. CSV file containing the Rule-based Question-Answering (QA) prompts used for impairment quantification. Each column is described in Table E of S1 Appendix.
(CSV)

S2 Data Chain-of-thought prompts. CSV file containing the Chain-of-Thought (CoT) prompts used for impairment quantification, following the same column format as S1 Data.
(CSV)
