# Supplementary material for: Vision-language models for human motion understanding: Lessons from stroke rehabilitation
Source: PLOS Digit Health. 2026 Jul 6;5(7):e0001506. doi: 10.1371/journal.pdig.0001506 (PMC13336467; doi:10.1371/journal.pdig.0001506)
Supplement: S1 Appendix — Supporting Information for the main text: choice of dataset split (Section A); VLM input preprocessing (Section B); more details on the activity videos (Section C); the PRIM-RS method (Section D); prompts for activity identification (Section E); direct vs. optimized prompting for activity identification (Section F); prompts for dose quantification (Section G); further results for dose quantification (Section H); the Fig 4(d) experimental procedure (Section I); more details on the Fugl-Meyer assessment videos (Section J); and prompts for impairment quantification (Section K). (PDF) [file pdig.0001506.s001.pdf]

# Supporting information

## Contents


A. Choice of dataset split

B. VLM Input Preprocessing

C. More Details on the Activity Videos

D. PRIM-RS Method

E. Prompts for Activity Identification

F. Direct vs. Optimized Prompting for Activity Identification

G. Prompts for Dose Quantification

H. Further Results for Dose Quantification

I. Fig 4(d) Experimental Procedure

J. More Details on the Fugl-Meyer Assessment Videos

K. Prompts for Impairment Quantification


## A Rationale for cohort selection

Here, we provide a justification for the selection of the individuals in each cohort in Table 2.

We designed the test cohort for Task A to be as inclusive as possible, evaluating activity identification across 640 videos from all available subjects outside the PO set. Because activity identification relies primarily on scene-level cues rather than fine-grained movement quality, only two control subjects were needed for prompt optimization.

Task B addresses dose quantification under two complementary conditions. Task B.1 serves as an exploratory study, evaluating 15 VLMs on all nine activities. Due to the computational cost (for a typical video of duration 52.1-s, evaluation is 97.7x more expensive as compared to Task A), the test cohort was limited to 10 subjects (90 videos). We excluded severe stroke subjects, including only 5 control and 5 mildly/moderately impaired subjects, because the atypical movement patterns of severe patients makes dose quantification particularly challenging (see, e.g. Table 3 in [27]). Task B.2 takes a more targeted approach: we focused on two of the nine activities—RTT and shelf—to investigate whether an optimized VLM-based system can achieve strong performance for these highly structured tasks (132 videos; 4 subjects did not complete the shelf task). We did not do experiments with additional VLMs, which resulted in a significantly lower computational cost than in Task B.2, which enabled us to include severe patients. When performing prompt optimization, we decided to include a severe stroke subject alongside two control subjects in the B.2 PO set, as a representative of a subject with abnormal motion. Unlike Task A, dose quantification is inherently movement-based, and the characteristics of the subject cohort directly influence the quality of the optimized prompts.

Task C evaluates impairment quantification across 899 videos, each corresponding to a manually segmented clip of a single Fugl-Meyer item. To minimize annotation effort while maintaining adequate coverage of the 0–66 FMA score range, we randomly sampled half of the impaired subjects and 4 of 18 controls, prioritizing impaired subjects to maintain adequate coverage of the 0–66 FMA score range.

## B VLM Input Preprocessing

We performed our evaluations with greedy decoding (temperature of 0, top-p set to None, and a beam size of 1). Table A shows hyperparameter configurations for the model families.

**Table A. Model Configurations.** We used the default model configurations provided by lmms_eval and list important settings here.

| Model Family | Configuration / Settings | Model Tags (Hugging Face Hub) |
|---|---|---|
| LLaVA-NeXT-Video [1] | conv_template=qwen_1_5[a] | lmms-lab/LLaVA-NeXT-Video-7B-Qwen2<br>lmms-lab/LLaVA-NeXT-Video-72B-Qwen2 |
| LLaVA-OneVision [2] | conv_template=qwen_1_5[a]<br>model_name=llava_qwen | lmms-lab/llava-onevision-qwen2-0.5b-ov<br>lmms-lab/llava-onevision-qwen2-7b-ov<br>lmms-lab/llava-onevision-qwen2-72b-ov-sft |
| NVILA [3] | conv_template=auto[a] | Efficient-Large-Model/NVILA-8B<br>Efficient-Large-Model/NVILA-15B |
| Qwen2.5-VL [4] | qwen-vl-utils: v0.0.11[b]<br>min_pixels: 256*28*28<br>max_pixels: 1,605,632 | Qwen/Qwen2.5-VL-7B-Instruct<br>Qwen/Qwen2.5-VL-32B-Instruct<br>Qwen/Qwen2.5-VL-72B-Instruct |
| InternVL3 / 3.5 [5,6] | modality=video<br>input_size=448[c]<br>max_num=1[d]<br>use_thumbnail=True[e] | OpenGVLab/InternVL3-78B<br>OpenGVLab/InternVL3_5-2B<br>OpenGVLab/InternVL3_5-8B<br>OpenGVLab/InternVL3_5-38B<br>OpenGVLab/InternVL3_5-30B-A3B |

[a] **conv_template**: Conversation template.
[b] **input_size**: The pre-processing library, qwen−vl−utils was updated in version 0.0.14 to resize based on min_pixels and max_pixels (unlike version 0.0.11, used in this study).
[c] **input_size**: The resolution (448x448) to which each image patch is resized.
[d] **max_num**: The maximum number of patches to split each frame into.
[e] **use_thumbnail**: Whether to include a downsampled thumbnail of the entire frame as an additional patch.

**Qwen2.5-VL Pre-processing** The pre-processing pipeline for Qwen2.5-VL does very minor reshaping. When given a video of eight frames at $704 \times 1088$ resolution, it proceeds as follows:

1. **Divisibility Resizing:** The frames are first resized to $\mathbf{700} \times \mathbf{1092}$. This is a minor adjustment to ensure both the height (700) and width (1092) are divisible by 28. This factor is derived from the spatial patch size ($p = 14$) and temporal patch size ($\tau = 2$).
2. **Pixel Constraint Check:** The min_pixels and max_pixels configurations in Table A are per-frame limits. The new resolution ($700 \times 1092 = 764,400$ pixels) is comfortably within this range, so no further resizing is needed to meet these constraints.
3. **Patching and Tokenization:** The video tensor, now with shape $(T, C, H, W) = (8, 3, 700, 1092)$, is converted into patch tokens.

- The $T = 8$ frames are grouped temporally by $\tau = 2$, resulting in **4 temporal groups**.
- Each frame is divided spatially into $p \times p = 14 \times 14$ patches.
- This yields $(H/p) \times (W/p) = (700/14) \times (1092/14) = 50 \times 78 = \mathbf{3,900}$ **spatial patches** per temporal group.
- The total number of initial patches (or "tubelets") sent to the visual encoder is 4 (groups) $\times\, 3,900$ (patches/group) $= \mathbf{15,600}$.

4. **Token Merging:** After the visual encoder, a patch merger module reduces the number of tokens by a factor of 4.

5. **Final Token Count:** The total number of pure vision tokens produced by this process is 15,600 / 4 = **3,900** tokens. These tokens are fed into the LLM, along with the prompt tokens.

## C More Details on the Activity Videos

Here, we provide visualizations into dataset statistics, as well as further details on each activity. See Fig A and Table B.

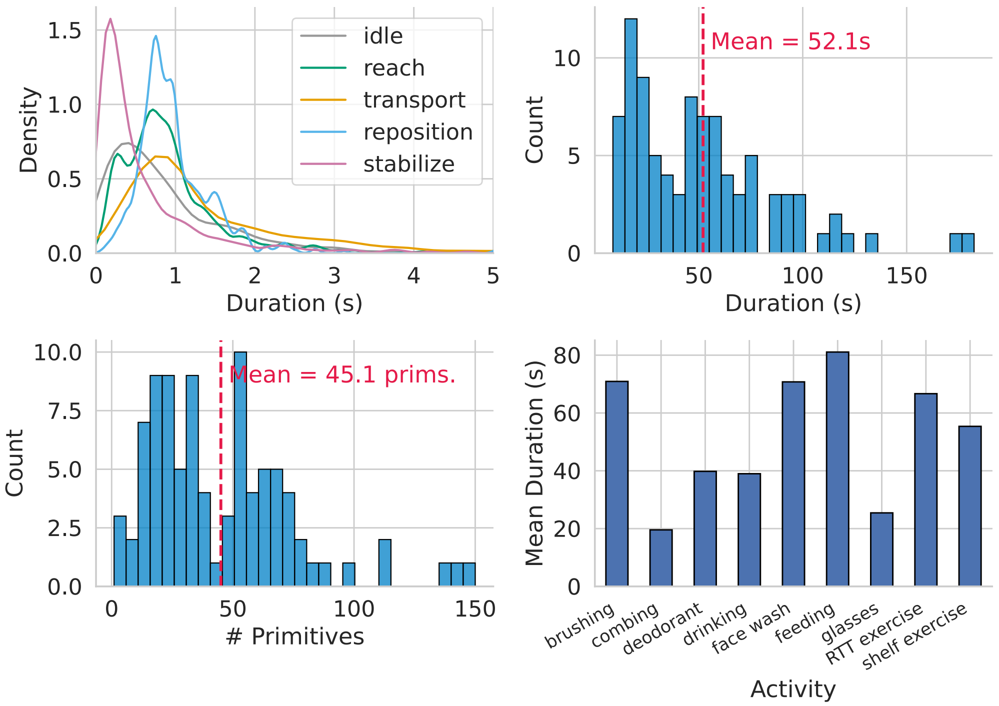


**Fig A. Dataset statistics for the 90-video data set used for primitives identification evaluation. Top left:** Kernel density estimate of primitive durations across all videos. **Top right:** Histogram of the video durations. **Bottom left:** Histogram of the number of primitives per video. **Bottom right:** Mean video durations across the nine activities.

**Table B.** Description of the activities performed by the stroke-impaired patients in the cohort. Sourced from [27].

| Activity | Workspace | Target object(s) | Instructions |
|---|---|---|---|
| Washing face | Sink with a small tub (32.3 x 24.1 x 2.5 $cm^3$) in it and two folded washcloths on either side of the countertop, 30 cm from edge closest to patient | Washcloths, faucet handle, and tub | Fill tub with water, dip washcloth on the right side into water, wring it, wiping each side of their face with wet washcloth, place it back on countertop. Use washcloth on the left side to dry face, place it back on countertop |
| Applying deodorant | Tabletop with deodorant placed at midline, 25 cm from edge closest to patient | Deodorant (solid twist-base) | Remove cap, twist base a few times, apply deodorant, replace cap, untwist the base, put deodorant on table |
| Hair combing | Tabletop with comb placed at midline, 25 cm from edge closest to patient | Comb | Pick up comb and comb both sides of head |
| Don/doffing glasses | Tabletop with glasses placed at midline, 25 cm from edge closest to patient | Pair of glasses | Wear glasses, return hands to table, remove glasses and place on table |
| Eating | Table top with a standard-size paper plate (21.6 cm diameter) placed at midline, 2 cm from edge, utensils placed 3 cm from edge, 5 cm from either side of plate, a baggie with a slice of bread placed 25 cm from edge, 23 cm left of midline, and a margarine packet placed 32 cm from edge, 17 cm right of midline | Paper plate, fork, knife, re-sealable sandwich baggie, slice of bread, single-serve margarine container | Remove bread from plastic bag and put it on plate, open margarine pack and spread it on bread, cut bread into four pieces, cut off and eat a small bite-sized piece |
| Drinking | Tabletop with water bottle and paper cup 18 cm to the left and right of midline, 25 cm from edge closest to patient | Water bottle (12 oz), paper cup (4 oz) | Open water bottle, pour water into cup, take a sip of water, place cup on table, and replace cap on bottle |
| Tooth brushing | Sink with toothpaste and toothbrush on either side of the countertop, 30 cm from edge closest to patient | Travel-sized toothpaste, toothbrush with built-up foam grip, faucet handle | Wet toothbrush, apply toothpaste to toothbrush, replace cap on toothpaste tube, brush teeth, rinse toothbrush and mouth, place toothbrush back on countertop |
| Moving object on a horizontal surface | Horizontal circular array (48.5 cm diameter) of 8 targets (5 cm diameter) | Toilet paper roll wrapped in self-adhesive wrap | Move the roll between the center and each outer target, resting between each motion and at the end |
| Moving object on/off a Shelf | Shelf with two levels (33 cm and 53 cm) with 3 targets on both levels (22.5 cm, 45 cm, and 67.5 cm away from the left-most edge) | Toilet paper roll wrapped in self-adhesive wrap | Move the roll between the center target and each target on the shelf, resting between each motion and at the end |

## D PRIM-RS Method

<u>Optimized Prompting:</u> We adapted the two binary prompts used in *Decomposed Prompting* as follows. First, we replaced motion detection with idle detection. Rather than asking whether the hand is in motion, the prompt queried whether the hand is still and not interacting with any object. This change was motivated by the observation that the VLM reliably detects idle hands, particularly during the repetitive act-and-rest

cycles characteristic of the RTT and shelf tasks, and it generally performed well in practice. Second, we modified grasp detection by introducing contextual prompting: the prompt asked whether the subject was picking up an object if the hand had been empty in the prior state, or releasing an object if one had been previously held. Contextual prompting yielded mixed results, and careful post-processing proved more critical for robust performance. Full prompt details are provided in Section G.

Pose-Informed Decision Making: PRIM-RS utilized a pose model for hand-region cropping, following the procedure detailed in the Cropping subsection. State assignment for each segment was handled as follows: if the cropping system abstained, the idle state was set to "idle" and the grasp state to "empty." If cropping succeeded and quick movement was detected, the idle state was set to "not idle" and the grasp state was retained from the previous segment, avoiding potentially inaccurate VLM predictions during rapid motion. Otherwise, we prompted the VLM to estimate both idle and grasp states. Concatenating these states resulted in an idle and grasp signal for the video.

Post-Processing: Post-processing was executed by sequentially filling an empty list with a length equal to the number of segments in the video, addressing the primitives in the following order: *idle*, *reach* and *reposition*, and *transport* and *stabilize.*

- *Idle:* Segments for the idle signal were relabeled in two passes: first to "not idle" if adjacent segments were "not idle," and then to "idle" if adjacent segments were "idle," using the updated labels from the first pass. Using the resulting smoothed idle signal, we corrected the grasp signal by setting any segment to "empty" whenever the corresponding idle state was "idle." We then applied the same smoothing operation to this updated grasp signal. Because such smoothing can mask short-duration events, we reduced the segment length from 0.533 s to 0.267 s (using four frames instead of eight) while maintaining the same frame rate.

- *Reach and reposition:* We labeled *reach* and *reposition* for all "not idle" and "empty-handed" segments. Consecutive segments were grouped into contiguous blocks, and each block was classified based on whether its surrounding segments were "idle" or "holding," yielding four possibilities. Following Table 3, each case was assigned one of *reach*, *reposition*, *reach-reposition*, or *reposition-reach.* Further, because direct idle–grasp or grasp–idle transitions were rare, we explicitly inserted *reach* or *reposition* where appropriate.

- *Transport and stabilize:* Finally, *transport* and *stabilize* were assigned within "holding" blocks. We detected stillness similarly to how quick movement was detected for cropping, but used $\leq 3$ px instead of $\geq 15$ px as the threshold. If, for a given block, stillness was detected for at least three consecutive segments, indicating confidence by the pose model, those three segments would be labeled as *stabilize.* Further, if stillness was detected within three segments of the block's end, that segment and the following segments within the block were labeled as *stabilize.* Other "holding" segments defaulted to *transport.*

## E Prompts for Activity Identification

Listed below are the direct and tailored prompts for activity identification, respectively.

**Listing A.** Activity Identification Direct Prompt

```
{
  "question": "Which activity is the patient performing in this
      video?",
```

```
  "response_instructions": "After noting your observations, end your reply with exactly one line: FINAL_ANSWER: <activity_name>",
  "instructions": "Determine the main activity being performed by the patient. Choose the most fitting activity label from the list below. Always respond with an activity, even if uncertain.",
  "activity_classes": {
    "Brushing": "The patient applies toothpaste to a toothbrush, brushes their teeth, rinses, and sets the brush back down.",
    "Combing": "The patient picks up a comb and combs both sides of their hair.",
    "Deodorant": "The patient twists open a deodorant stick, applies it under the arm, then replaces the cap.",
    "Drinking": "The patient pours water from a bottle into a cup, takes a sip, and replaces the cap.",
    "Face wash": "The patient washes and dries their face using two washcloths at a sink.",
    "Feeding": "The patient prepares bread with margarine on a plate and eats a small piece using utensils.",
    "Glasses": "The patient puts on or removes a pair of glasses from the tabletop.",
    "RTT exercise": "The patient slides a toilet paper roll between center and outer targets on a flat surface.",
    "Shelf exercise": "The patient transfers a toilet paper roll between the center target and multiple shelf levels."
  }
}
```

**Listing B.** Activity Identification Tailored Prompt

```
{
  "question": "Which activity is the patient performing in this video?",
  "response_instructions": "After noting your observations, end your reply with exactly one line: FINAL_ANSWER: <activity_name>",
  "instructions": "Determine the main activity being performed by the patient. Choose the most fitting activity label from the list below. Always respond with an activity, even if uncertain.",
  "activity_classes": {
    "Brushing": "The patient is at the sink. Either toothpaste or a toothbrush is visible, indicating the patient is brushing their teeth.",
    "Combing": "The patient grabs a small rectangular object (likely a comb) on the table and moves it near the hair area (likely to groom their hair).",
    "Deodorant": "The patient applies deodorant using a deodorant tube (likely white) on the table. The hand is seen moving towards the underarm area.",
```

```
    "Drinking": "The patient pours water from a plastic water
        bottle into a cylindrical cup on the table and takes a
        sip.",
    "Face wash": "The patient washes their face at the sink
        using water and a wash cloth or towel.",
    "Feeding": "The patient prepares and eats bread on a white
        plate by spreading margarine, cutting it, and taking
        bites.",
    "Glasses": "The patient grabs a pair of glasses from the
        table and puts them on or removes them.",
    "RTT exercise": "The patient repeatedly moves a cylindrical
         block on the table. ",
    "Shelf exercise": "The patient repeatedly moves a
        cylindrical block on a TRANSPARENT shelf.",
  }
}
```

## F Direct vs. Optimized Prompting for Activity Identification

Fig B compares direct and optimized prompting for activity identification across all 640 videos (healthy controls and stroke patients combined). Direct prompting, based on a pre-existing description of the activities, achieves 53.4% accuracy—substantially better than predicting the majority class (13.4%), but limited by frequent misclassifications of combing, deodorant, and shelf activities as RTT, likely because these activities share the same workspace. Optimized prompting, tailored to Qwen2.5-VL-7B-Instruct using videos from two held-out control subjects, improves accuracy to 77.5%. See the main text for control-vs.-stroke breakdowns for the optimized prompt.

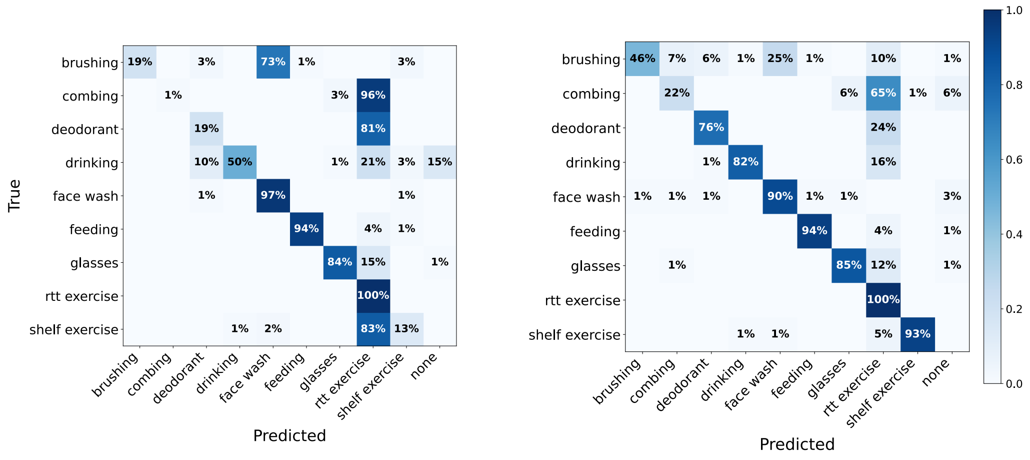


**Fig B. Direct vs. optimized prompting for activity identification.** Confusion matrices for Qwen2.5-VL-7B-Instruct across all $N$=640 videos (controls and stroke patients combined). Each cell shows the fraction of samples from a true activity (row) predicted as a given activity (column). **Left:** Direct prompting (53.4% accuracy). **Right:** Optimized prompting (77.5% accuracy). Optimized prompting substantially reduces confusion, particularly for combing, deodorant, and shelf activities that were frequently misclassified as RTT under direct prompting.

## G Prompts for Primitive Identification

Here, we list the prompts used in our experiments for primitive identification. We first tested an *Ideal Dose Quantification Prompt* that asks for a sequence of primitives per segment, whose poor performance led to the *Single-Prediction Dose Quantification Prompt*. Afterwards, we tested *Decomposed Prompting* and a *Contextual Prompting*. Finally, refer to *PRIM-RS Prompts* for the RTT/shelf-specific pipeline.

For each prompt, we chose the text within the parentheses, separated by "—", to cater to the specific situation. For contextual prompting, we additionally chose the hand reference (e.g. "hand in the center" vs. "patient's LEFT hand") based on whether cropping was successful.

**Listing C.** Ideal Dose Quantification Prompt

```
Focus on the patient's (LEFT|RIGHT) hand. Output the sequence
    of functional primitives performed by the patient's (LEFT|
    RIGHT) hand as a comma-separated list.

Functional primitives:
- IDLE: hand is waiting
- REACH: hand in motion with the purpose of contact with an
    object
- REPOSITION: hand in motion with no contact at the endpoint
- STABILIZE: hand steady to keep a target object still
- TRANSPORT: hand in motion to convey an object in space
Only output the functional primitives (no definitions) as a
    comma-separated list.
```

**Listing D.** Single-Prediction Dose Quantification Prompt

```
Focus on the patient's (LEFT|RIGHT) hand. Output the functional
    primitive performed by the patient's (LEFT|RIGHT) hand as
    a single word.

Functional primitives:
- IDLE: hand is waiting
- REACH: hand in motion with the purpose of contact with an
    object
- REPOSITION: hand in motion with no contact at the endpoint
- STABILIZE: hand steady to keep a target object still
- TRANSPORT: hand in motion to convey an object in space
Only output one functional primitive.
```

**Listing E.** Decomposed Prompting (Motion) (1/2)

```
Focus on the patient's (LEFT|RIGHT) hand. Is it actively moving
    an object, moving towards an object, or moving away from
    an object? Answer YES or NO.
```

**Listing F.** Decomposed Prompting (Grasp) (2/2)

```
Focus on the patient's (LEFT|RIGHT) hand. Is it actively
    grasping or holding an object? Answer YES or NO.
```

**Listing G.** Contextual Prompting (Motion) (1/2)

```
Focus on the (hand in the center|patient's LEFT hand|patient's
    RIGHT hand). It was previously (still|moving an object or
    moving toward/away from one). Is it now (actively moving an
     object, moving towards an object, or moving away from an
    object|still)? Answer YES or NO.
```

**Listing H.** Contextual Prompting (Grasp) (2/2)

```
Focus on the (hand in the center|patient's LEFT hand|patient's
    RIGHT hand). Previously, (it was actively grasping an
    object|the hand was empty). Does it (release the object|
    grasp an object) in this clip? Answer YES or NO directly.
```

**Listing I.** PRIM-RS Prompts (Idle) (1/3)

```
Is (the hand|the patient's LEFT hand|the patient's RIGHT hand)
    idle in this video clip?
(Idle) Visibly resting on the black mat, not moving, and not
    interacting with a cylindrical block. (Active) In the air,
    moving towards an object, moving away from an object,
    interacting with a cylindrical block, or 'resting' on a
    cylindrical block. The hand can be moving very slowly
    through the air and still be considered 'active.' Answer '
    Yes.' if (the hand|the patient's LEFT hand|the patient's
    RIGHT hand) is idle; answer 'No.' otherwise.
```

**Listing J.** PRIM-RS Prompts (Grasp) (2/3)

```
This is a chunk from a video sequence. In the previous chunk, (
    the hand|the patient's LEFT hand|the patient's RIGHT hand)
    was not holding anything. Answer directly: 'Yes.' if (the
    hand|the patient's LEFT hand|the patient's RIGHT hand)
    visibly picks up a cylindrical block in this chunk; answer
    'No.' otherwise.
Mere contact does not count as grasping.
```

**Listing K.** PRIM-RS Prompts (Release) (3/3)

```
This is a chunk from a video sequence. In the previous chunk, (
    the hand|the patient's LEFT hand|the patient's RIGHT hand)
    was holding a cylindrical block. Answer directly: 'Yes.' if
     (the hand|the patient's LEFT hand|the patient's RIGHT hand
    ) puts down and releases the block; answer 'No.' otherwise.
```

Listing I (idle vs. active framing) reflects that VLMs are more reliable detectors of "idle" than of "movement." This is a property of the model rather than our PO subjects. The sentence about slow motion through the air was added due to observations of the VLM outputs, leading us to clarify that slow drift through the air should be classified as active (not idle) by definition. Listings J and K introduce a stateful grasp/release formulation targeted at the RTT and shelf tasks, with explicit reference to the cylindrical rehab exercise object used in those tasks.

# H Further Results for Dose Quantification

Table C shows ablation results for prompting the primitive directly, as in Listing D. Table D shows results for using *Decomposed Prompting* (see Listings E and F for the prompts). We find that the performance of the two prompting methods to be comparable, and proceeded with *Decomposed Prompting* in the main section to provide more granular analyses.

**Table C. Ablations for Direct Prompting using the prompt in Listing D.**

| $f$ | $n$ | ES ↑ | AER ↓ | RCE ↓ |
|---|---|---|---|---|
| 1 | 1 | 41.83 ± 1.01 | 0.65 ± 0.04 | 0.65 ± 0.04 |
| 2 | 2 | 41.25 ± 1.07 | 0.64 ± 0.02 | 0.64 ± 0.03 |
| 4 | 4 | 41.87 ± 1.19 | **0.62 ± 0.02** | **0.60 ± 0.03** |
| 8 | 8 | 40.40 ± 1.21 | 0.65 ± 0.03 | 0.64 ± 0.03 |
| 15 | 15 | 41.86 ± 1.20 | **0.61 ± 0.02** | **0.63 ± 0.03** |
| 30 | 30 | 40.39 ± 1.24 | 0.63 ± 0.02 | 0.68 ± 0.02 |
| 2 | 1 | 44.24 ± 1.01 | 0.81 ± 0.08 | 0.82 ± 0.08 |
| 4 | 2 | 44.92 ± 1.00 | 0.82 ± 0.06 | 0.81 ± 0.06 |
| 8 | 4 | **45.70 ± 1.02** | 0.69 ± 0.04 | 0.68 ± 0.04 |
| 15 | 8 | **46.34 ± 0.97** | 0.69 ± 0.05 | 0.71 ± 0.05 |
| 30 | 15 | 43.73 ± 1.09 | 0.72 ± 0.05 | 0.77 ± 0.05 |
| 4 | 1 | 38.56 ± 1.19 | 1.44 ± 0.14 | 1.45 ± 0.14 |
| 8 | 2 | 37.81 ± 1.10 | 1.48 ± 0.13 | 1.46 ± 0.13 |
| 15 | 4 | 40.25 ± 1.12 | 1.25 ± 0.11 | 1.28 ± 0.10 |
| 30 | 8 | 41.39 ± 1.08 | 1.07 ± 0.07 | 1.08 ± 0.07 |
| 8 | 1 | 28.44 ± 1.11 | 2.73 ± 0.24 | 2.73 ± 0.24 |

**Table D. Ablations for *Decomposed Prompting* using the prompts in Listings E and F.**

| $f$ | $n$ | ES ↑ | AER ↓ | RCE ↓ |
|---|---|---|---|---|
| 1 | 1 | 36.89 ± 1.63 | 0.68 ± 0.03 | 0.68 ± 0.03 |
| 2 | 2 | 34.52 ± 1.73 | 0.70 ± 0.03 | 0.70 ± 0.03 |
| 4 | 4 | 35.87 ± 1.70 | 0.67 ± 0.02 | 0.67 ± 0.02 |
| 8 | 8 | 37.45 ± 1.64 | 0.68 ± 0.04 | 0.67 ± 0.04 |
| 15 | 15 | 42.52 ± 1.47 | **0.60 ± 0.02** | **0.61 ± 0.03** |
| 30 | 30 | 42.71 ± 1.39 | **0.62 ± 0.04** | **0.64 ± 0.04** |
| 2 | 1 | 42.80 ± 1.64 | 0.73 ± 0.08 | 0.73 ± 0.08 |
| 4 | 2 | 40.68 ± 1.74 | 0.74 ± 0.08 | 0.75 ± 0.08 |
| 8 | 4 | 44.16 ± 1.75 | 0.71 ± 0.08 | 0.70 ± 0.08 |
| 15 | 8 | 46.69 ± 1.62 | 0.65 ± 0.06 | 0.65 ± 0.06 |
| 30 | 15 | **49.21 ± 1.39** | 0.64 ± 0.08 | 0.65 ± 0.08 |
| 4 | 1 | 45.64 ± 1.68 | 0.93 ± 0.17 | 0.97 ± 0.17 |
| 8 | 2 | 44.57 ± 1.67 | 0.94 ± 0.17 | 1.00 ± 0.17 |
| 15 | 4 | 48.22 ± 1.69 | 0.86 ± 0.15 | 0.88 ± 0.15 |
| 30 | 8 | **50.76 ± 1.55** | 0.82 ± 0.13 | 0.84 ± 0.13 |
| 8 | 1 | 42.42 ± 1.64 | 1.45 ± 0.29 | 1.53 ± 0.28 |

The tables also compare different settings of the sampling rate ($f$) and number of frames per segment ($n$), sorted in decreasing segment duration. There is a trade-off between the three metrics: with decreasing segment duration, sequence predictions

become more granular but also longer. This trend is reflected by a generally improving ES, and worsening AER and RCE. For *Decomposed Prompting,* the best trade-off appears to occur with segment duration 0.533 seconds. We chose the setting of $f = 15$ and $n = 8$ as a good balance of performance and compute speed for our model ablations.

To complement the sequence-level results in Fig 4, which showed that VLMs lack the precision needed for dose quantification in general activities, we present primitive-level confusion matrices in Fig C. Two patterns emerge. First, the model is systematically biased toward positive predictions on both the grasp and motion dimensions. This bias manifests in several ways: it under-predicts the *idle* class, misclassifies *reach* as *transport* (hallucinating a grasp), misclassifies *stabilize* as *transport* (hallucinating motion), and — consistent with these tendencies — achieves high accuracy on *transport*, where both grasp and motion are truly present. Second, the model frequently misclassifies *reposition* as *idle*, suggesting it confuses a hand moving near its resting position with a hand truly at rest.

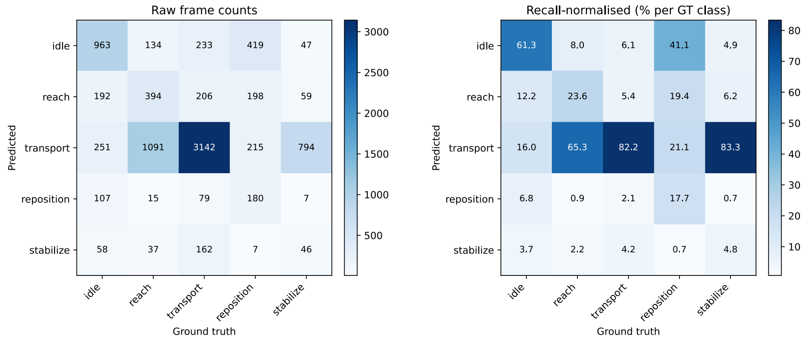


**Fig C. Primitive-level recognition results. (Left)** The primitive-level confusion matrix following the same evaluation procedure as Fig 4. Each unit corresponds to one video segment. **(Right)** The confusion matrix normalised by column.

## I Fig 4(d) Experimental Procedure

For Fig 4(d), we sourced videos from 5 control subjects. These subjects performed the RTT task twice, once for either hand, filmed via two camera streams positioned at the front-left and front-right of the person (making 20 videos in total). Crucially, the RTT task was designed so that only one hand performs the RTT task while the other hand remains still, with the first hand labeled. We chose segments where every frame is labeled with a moving primitive, i.e. one of *transport*, *reach*, or *reposition*, leading to 1,101 segments of duration 0.533 seconds. We then evaluated two methods for motion prediction on these segments: **Pred w/o Cropping**, where we prompted the model twice, once for each hand, asking if the hand was moving; and **Pred w/ Cropping**, where we tested if we could improve results by cropping around the desired hand and asking about the movements for the "hand in the center." The prompts for both scenarios are listed below.

**Listing L.** Cross-hand Prompting (Pred w/o Cropping) (1/2)

```
Focus on the patient's (LEFT/RIGHT) hand. Do not mention or
    consider the other hand in any way. Based on the movement
    and posture of the patient's (LEFT/RIGHT) hand, is the (
    LEFT/RIGHT) hand moving or moving an object? Answer 'Yes.'
    or 'No.' directly.
```

**Listing M.** Cross-hand Prompting (Pred w/ Cropping—Even if Cropping Fails) (2/2)

```
Based on the movement and posture of the hand, is the hand in
    the center moving or moving an object? Answer 'Yes.' or 'No
    .' directly.
```

## J More Details on the Fugl-Meyer Assessment Videos

Here, we illustrate the two views for the Fugl-Meyer assessment (Figs D and E), and justify the short video duration for videos not in the **Speed/Coord.** section (see Fig F).

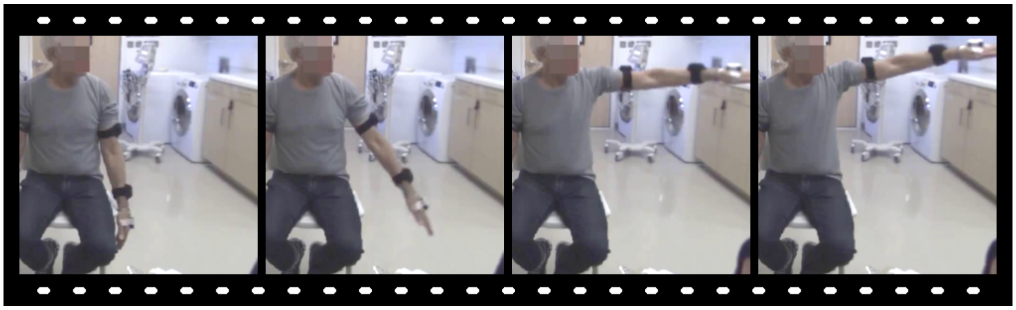

**Fig D. Front view for the Fugl-Meyer assessment.**

## K Prompts for Impairment Quantification

We include the prompts for **Rule-based Question-Answering** and **Chain-of-Thought** as supplementary CSV files, S1 Data and S2 Data, respectively. Table E describes each column in the files.

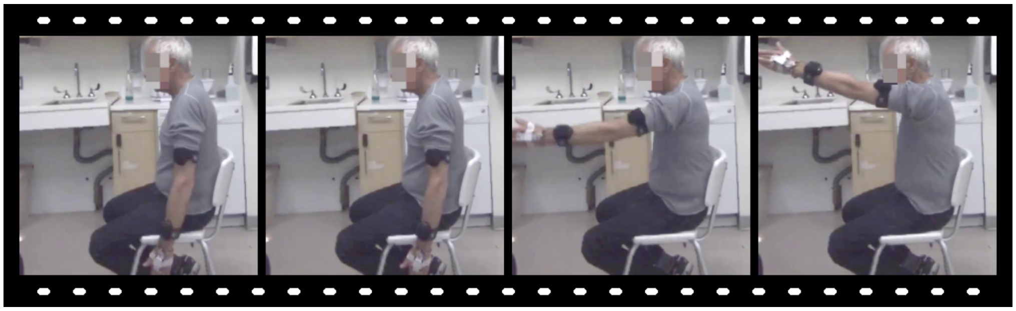

**Fig E. Side view for the Fugl-Meyer assessment. (Different assessment item from Fig D)**

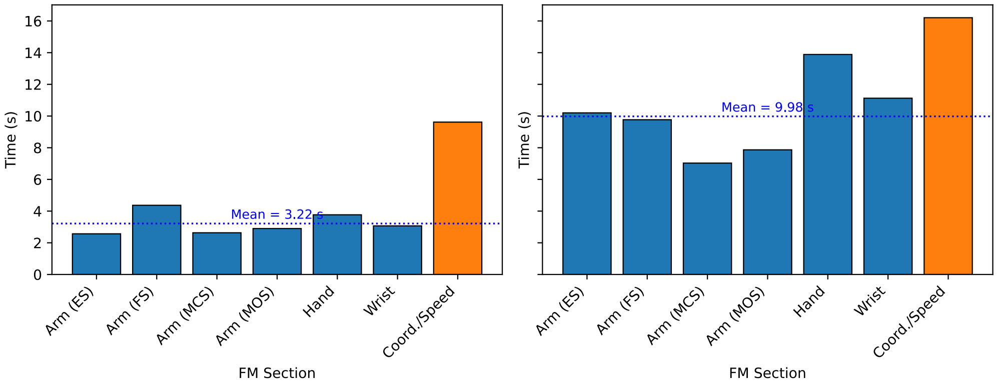


**Fig F. Duration of Fugl-Meyer assessment videos by item section. Left:** Median video duration in seconds. **Right:** 95-percentile video duration in seconds. The means are calculated using the videos in all sections except **Coord./Speed.**

**Table E. Description of the columns in the prompt CSV files**

| Column | Description |
|---|---|
| `qid` | Question identifier (integer index). |
| `fm_video` | Video identifier in the format {`fm_item`}_{`side`}_{`view`}. `fm_item` ranges from 3 to 33 and denotes the Fugl–Meyer (FM) assessment item. `side` is either `A` (affected) or `H` (healthy); most rows use `A`, but `H` is included for Coordination/Speed items to compare movement between sides. `view` indicates the camera view that best answers the question: `F` (front) or `S` (side). |
| `question_type` | Type of question, either `rate` (requires a numerical rating) or `binary` (yes/no response). |
| `sampling` | Frame sampling method. `uniform`: sample 8 frames uniformly across the video. `dense`: segment the video into 0.267 s chunks and sample 8 frames uniformly within each chunk. |
| `binary_no_score` | For binary questions, if non-null, specifies the score to assign when the VLM predicts "no." If null, the question chain continues. |
| `binary_yes_score` | For binary questions, analogous to `binary_no_score`, but specifies the score to assign when the VLM predicts "yes." |
| `question` | The natural-language prompt given to the VLM (e.g., "Assess the patient's maximal shoulder elevation at the ending position. Rate as follows: 0 (no elevation), 1 (partial elevation), or 2 (full elevation). Answer directly."). |
